# Genomic and Transcriptomic Approaches Advance the Diagnosis and Prognosis of Neurodegenerative Diseases

**DOI:** 10.3390/genes16020135

**Published:** 2025-01-24

**Authors:** Zheng Liu, Si-Yuan Song

**Affiliations:** 1 Pathology Department, University of Texas MD Anderson Cancer Center, Houston, TX 77030, USA; zhengliu.jan@gmail.com; 2 Department of Neuroscience, Baylor College of Medicine, Houston, TX 77030, USA

**Keywords:** neurodegenerative diseases, genomics approaches, transcriptomics, biomarkers, diagnosis, prognostic trajectories

## Abstract

Neurodegenerative diseases, such as Alzheimer’s disease (AD), Parkinson’s disease (PD), Huntington’s disease (HD), and amyotrophic lateral sclerosis (ALS), represent a growing societal challenge due to their irreversible progression and significant impact on patients, caregivers, and healthcare systems. Despite advances in clinical and imaging-based diagnostics, these diseases are often detected at advanced stages, limiting the effectiveness of therapeutic interventions. Recent breakthroughs in genomic and transcriptomic technologies, including whole-genome sequencing, single-cell RNA sequencing (scRNA-seq), and CRISPR-based screens, have revolutionized the field, offering new avenues for early diagnosis and personalized prognosis. Genomic approaches have elucidated disease-specific genetic risk factors and molecular pathways, while transcriptomic studies have identified stage-specific biomarkers that correlate with disease progression and severity. Furthermore, genome-wide association studies (GWAS), polygenic risk scores (PRS), and spatial transcriptomics are enabling the stratification of patients based on their risk profiles and prognostic trajectories. Advances in functional genomics have uncovered actionable targets, such as ATXN2 in ALS and TREM2 in AD, paving the way for tailored therapeutic strategies. Despite these achievements, challenges remain in translating genomic discoveries into clinical practice due to disease heterogeneity and the complexity of neurodegenerative pathophysiology. Future integration of genetic technologies holds promise for transforming diagnostic and prognostic paradigms, offering hope for improved patient outcomes and precision medicine approaches.

## 1. Introduction

**Figure 1:**
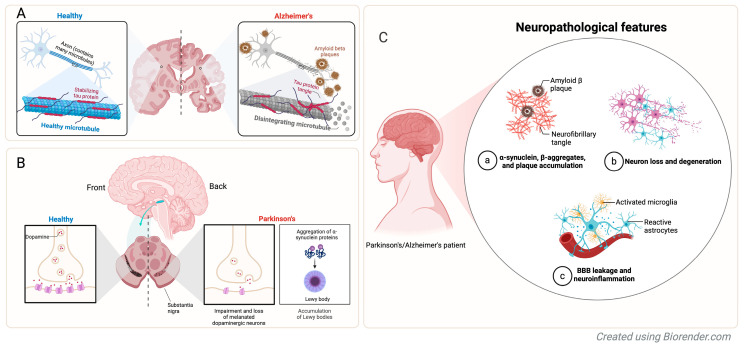
Neuropathological features of Alzheimer’s and Parkinson’s diseases. This figure illustrates the key neuropathological features of Alzheimer’s disease (AD) and Parkinson’s disease (PD) compared to healthy brain structures. (**A**) In AD, healthy microtubules stabilized by tau proteins (left) degenerate due to tau tangles and β-amyloid plaque formation (right), leading to disrupted neuronal function; (**B**) In PD, healthy dopaminergic neurons in the substantia nigra (left) are impaired by the aggregation of α-synuclein proteins into Lewy bodies (right), resulting in the loss of dopamine signaling and motor dysfunction; (**C**) Both diseases share common features such as (a) protein aggregates, such as α-synuclein, β-amyloid plaques, and neurofibrillary tangles, (b) progressive neuronal loss and degeneration, and (c) blood–brain barrier (BBB) leakage and neuroinflammation, which exacerbate neuronal damage and degeneration.

Neurodegenerative diseases, including Alzheimer’s disease (AD), Parkinson’s disease (PD), Huntington’s disease (HD), amyotrophic lateral sclerosis (ALS), and Lewy body dementia (LBD), represent some of the most pressing medical and societal challenges of our time [1,2]. These conditions progressively damage specific neuronal populations, leading to the deterioration of cognitive, motor, and emotional functions. For instance, Alzheimer’s disease is marked by β-amyloid plaque accumulation and neurofibrillary tangles (Figure 1A), while Parkinson’s disease is characterized by α-synuclein aggregates (Lewy bodies) within neurons, leading to progressive loss and degeneration of brain tissue (Figure 1B) [3,4]. Despite their distinct features, both AD and PD share common neuropathological aspects, such as blood–brain barrier (BBB) leakage, neuroinflammation, and subsequent neuronal loss (Figure 1C), further exacerbating disease progression [5,6]. With an aging global population, the prevalence of these conditions is rising sharply. AD affects nearly 30% of individuals over 85, while PD impacts approximately 1% of people over 60 [7,8,9]. These diseases follow an irreversible trajectory, ultimately resulting in severe disability and premature mortality. Patients often struggle with basic tasks, lose independence, and endure emotional distress, while caregivers face physical, emotional, and financial strain. Society bears the weight of these diseases as well, with healthcare systems strained by the high costs of long-term care and treatment. AD alone costs the U.S. over USD 345 billion annually, with numbers expected to escalate as the population ages [10].

Despite ongoing research, the diagnosis and prognosis of neurodegenerative diseases remain significant challenges. Current diagnostic techniques rely heavily on clinical evaluations, imaging modalities, and biomarker assessments, which often detect the disease only at advanced stages when neurodegeneration is already extensive [11,12]. For instance, AD is typically diagnosed when cognitive impairments are apparent and irreversible brain damage has occurred [9,13]. Similarly, PD diagnosis relies on motor symptoms, which manifest only after substantial neuronal loss in the substantia nigra [14,15]. Early detection is further complicated by overlapping symptoms among these diseases, such as cognitive decline in AD and LBD or motor dysfunction in PD and ALS [16].

Another limitation is the heterogeneity of these diseases. Genetic, environmental, and lifestyle factors contribute to diverse clinical manifestations, making it difficult to design universal diagnostic and therapeutic strategies [17]. For example, treatments effective in familial cases of ALS or HD, which have clear genetic markers, may not work for sporadic cases that lack such markers [18,19]. This variability necessitates personalized diagnostic and treatment approaches. Additionally, the lack of reliable biomarkers for disease progression and therapeutic response adds to the difficulty of predicting outcomes, further complicating prognosis and clinical management [20].

Recent advances in genomics have offered unprecedented opportunities to revolutionize the understanding, diagnosis, and prognosis of neurodegenerative diseases. Genomic approaches, such as whole-genome sequencing (WGS), transcriptomics, and epigenomics, have unveiled critical insights into the molecular underpinnings of these diseases. By identifying genetic risk factors, pathogenic mutations, and dysregulated pathways, these tools not only deepen our understanding of disease mechanisms but also facilitate the development of novel diagnostic biomarkers and therapeutic targets [1,21]. For instance, the identification of mutations in the *APP*, *PSEN1*, and *PSEN2* genes has provided a genetic basis for familial AD. These mutations disrupt amyloid precursor protein (APP) processing, leading to abnormal cleavage by β- and γ-secretases and subsequent amyloid plaque formation, a hallmark of AD (Table 1 and Supplementary Figure S1A) [22,23]. Similarly, variants in the *LRRK2*, *PARK2/PRNK,* and *SNCA* genes have advanced our understanding of familial PD. Dysregulation of α-synuclein homeostasis, resulting from *SNCA* mutations, contributes to protein misfolding, toxic oligomer accumulation, and Lewy body formation, exacerbated by mitochondrial dysfunction and oxidative stress (Table 1 and Supplementary Figure S1B) [24]. In addition to familial cases, genomic data have also helped uncover polygenic risk scores, enabling the stratification of individuals at risk of sporadic forms of these diseases. Additionally, emerging technologies such as single-cell RNA sequencing (scRNA-seq) and CRISPR-based gene editing are paving the way for precision medicine approaches, offering hope for earlier and more accurate diagnoses, as well as personalized therapeutic interventions [25].

In this review, we will explore the emerging role of genomic approaches in the diagnosis and prognosis of neurodegenerative diseases. We will discuss their utility in identifying genetic risk factors, predicting disease onset, and monitoring progression, as well as the challenges and future directions in integrating genomics into clinical practice.

## 2. Current Methods for the Diagnosis and Prognosis of Neurodegenerative Diseases

### 2.1. Clinical Evaluation and Neuropsychological Assessment

#### 2.1.1. Clinical Evaluation

Clinical evaluation remains a cornerstone in the diagnostic process for neurodegenerative diseases, offering a foundational understanding of symptom patterns, disease progression, and potential differential diagnoses. This process begins with a thorough assessment of the patient’s medical and family history, which can provide valuable diagnostic insights, especially in conditions with a genetic predisposition. Family member testing and segregation analysis are critical in such cases, as they allow clinicians to trace inheritance patterns, confirm the pathogenicity of identified variants, and assess the genetic risk for at-risk relatives. For example, familial cases of Huntington’s disease, caused by a CAG repeat expansion in the *HTT* gene, and ALS, linked to *SOD1* and *C9orf72* mutations, follow distinct inheritance patterns and present with characteristic motor or cognitive dysfunctions (Table 1) [27,31]. A detailed neurological and physical examination follows, focusing on motor, sensory, reflex, and coordination functions to identify disease-specific manifestations. For instance, hallmark symptoms like resting tremors, bradykinesia, rigidity, and postural instability are characteristic of PD, while AD often presents with cognitive decline, particularly memory impairment, alongside language and visuospatial deficits [32,33,34]. ALS is distinguished by the presence of both upper and lower motor neuron signs, such as muscle weakness, spasticity, and fasciculations, whereas Huntington’s disease frequently involves motor dysfunctions like chorea, accompanied by cognitive and psychiatric symptoms (Table 1) [35,36]. Despite its importance in detecting early and disease-specific signs, clinical evaluation alone often lacks the precision needed for a definitive diagnosis, as overlapping symptoms in the early stages of many neurodegenerative diseases complicate differentiation [37]. This highlights the necessity of integrating clinical assessments with advanced diagnostic tools to improve diagnostic accuracy and prognostic predictions.

#### 2.1.2. Neuropsychological Testing

Neuropsychological tests are used to assess cognitive functions, including memory, attention, language, executive function, and visuospatial skills. These tests help in identifying patterns of cognitive impairment associated with specific diseases and play a pivotal role in the diagnostic process for neurodegenerative diseases by offering a comprehensive assessment of symptoms and cognitive functions. Neuropsychological tools, such as the mini-mental state examination (MMSE) and the Montreal cognitive assessment (MoCA), and the clinical dementia rating (CDR) test are widely used to evaluate cognitive domains like memory, attention, language, executive function, and visuospatial skills [38,39,40]. These tests help identify cognitive impairment patterns associated with specific diseases, with the MMSE serving as a common tool for assessing global cognitive function and dementia, while the MoCA is particularly sensitive for detecting mild cognitive impairment (MCI), often a precursor to AD. The CDR test, on the other hand, provides a staging tool for dementia severity, offering a structured evaluation of memory, orientation, judgment, problem-solving, and daily function [41].

Neurophysiological examinations, such as electromyography (EMG) and nerve conduction studies, complement these evaluations by assessing the functional status of motor and sensory neurons, which is critical for diagnosing conditions like ALS, where motor neuron dysfunction is a hallmark feature [42]. Although these assessments provide essential diagnostic insights, they are often insufficient for revealing the structural or functional changes in the brain that underlie neurodegenerative processes. Consequently, neuroimaging and biomarker analysis are frequently employed alongside clinical evaluations to enhance diagnostic accuracy and uncover disease mechanisms.

### 2.2. Biomarker Analysis

Biomarker analysis provides critical insights into the molecular and cellular mechanisms underlying neurodegenerative diseases, serving as a valuable complement to clinical and neuroimaging assessments. Biomarkers can be obtained from cerebrospinal fluid (CSF), blood, or imaging studies, offering diagnostic and prognostic information.

#### 2.2.1. Cerebrospinal Fluid (CSF) Biomarkers

CSF biomarkers have become essential tools for diagnosing Alzheimer’s disease and other neurodegenerative disorders due to their ability to reflect pathological changes in the brain. Key biomarkers include β-amyloid (Aβ42), whose reduced levels in CSF signify amyloid plaque deposition, a hallmark of Alzheimer’s disease. Total tau (t-tau) and phosphorylated tau (p-tau) are also important indicators; elevated levels correspond to neuronal injury and tau pathology, respectively [43,44]. Beyond Alzheimer’s disease, abnormal α-synuclein levels in CSF have been associated with Parkinson’s disease and Lewy body dementia [45]. Despite their utility, the invasive nature of lumbar punctures required for CSF collection remains a limitation, and while these biomarkers provide detailed molecular insights, they often require complementary diagnostic methods to enhance specificity and sensitivity across different neurodegenerative diseases.

#### 2.2.2. Blood Biomarkers

Blood-based biomarkers are emerging as a promising alternative to CSF biomarkers, offering a less invasive method for detecting neurodegenerative diseases. Plasma β-amyloid and tau levels, for instance, are being actively investigated as diagnostic markers for Alzheimer’s disease. Similarly, neurofilament light chain (NfL), a marker of axonal damage, has shown potential in various conditions, including ALS, Alzheimer’s disease, and Parkinson’s disease [46]. The accessibility and scalability of blood biomarker testing make it an attractive option for widespread use; however, challenges remain regarding specificity and sensitivity, as some markers may overlap across multiple neurodegenerative conditions [47]. Further validation and integration with other diagnostic modalities are necessary to fully realize the potential of blood biomarkers in clinical practice.

#### 2.2.3. Saliva and Urine Biomarkers

Saliva and urine are gaining attention as accessible and non-invasive biofluids for biomarker discovery in neurodegenerative diseases. Saliva, rich in proteins and metabolites, offers potential for detecting biomarkers such as α-synuclein, tau, and β-amyloid, which may reflect underlying neurodegenerative processes. For instance, increased salivary α-synuclein has been associated with Parkinson’s disease, while salivary tau and β-amyloid levels are being studied for their relevance to AD [48,49]. Similarly, urine has been explored as a biofluid for detecting oxidative stress markers and metabolites linked to neurodegenerative diseases. For example, increased levels of urinary 8-hydroxy-2′-deoxyguanosine (8-OHdG) have been reported in patients with PD and ALS, indicating heightened oxidative stress [50,51].

Despite their promise, saliva and urine biomarkers face challenges that limit their widespread application in clinical practice. Salivary biomarker studies are often constrained by variability in sample collection, the presence of confounding factors such as oral health status, and relatively low biomarker concentrations compared to CSF or blood. Similarly, urinary biomarkers may be influenced by systemic metabolic factors and lack the specificity required to distinguish between neurodegenerative conditions. Further standardization of collection protocols and validation of biomarker candidates are needed to address these limitations and enhance their diagnostic utility.

### 2.3. Neuroimaging

Neuroimaging is a cornerstone in the diagnostic process for neurodegenerative diseases, offering non-invasive insights into structural, functional, and molecular brain changes. It serves as a critical tool for confirming diagnoses, monitoring disease progression, and identifying potential therapeutic targets.

#### 2.3.1. Structural Imaging (MRI and CT)

Magnetic resonance imaging (MRI) is widely used to assess structural abnormalities in the brain, providing high-resolution images that aid in the diagnosis of neurodegenerative diseases. In AD, hippocampal atrophy and cortical thinning observed on MRI are hallmarks of disease progression, though they are not exclusive to AD and may overlap with conditions like vascular dementia or frontotemporal dementia (FTD) [52]. In PD, neuromelanin-sensitive MRI identifies signal loss in the substantia nigra, while diffusion tensor imaging (DTI) assesses nigrostriatal pathway integrity, aiding early diagnosis and differentiation from atypical parkinsonian syndromes [53]. HD is characterized by striatal atrophy, particularly in the caudate nucleus and putamen, which MRI can quantify even before motor symptoms emerge [54]. Similarly, in ALS, MRI highlights corticospinal tract degeneration and motor cortex atrophy, and advanced techniques like magnetic resonance spectroscopy (MRS) can detect metabolic changes, such as reduced N-acetylaspartate levels [55].

However, MRI has notable limitations. While it is effective at identifying macroscopic structural changes, it lacks the sensitivity to detect early-stage molecular alterations or subtle differences in overlapping pathological features. Furthermore, it is time-consuming and costly compared to other imaging modalities. Computed tomography (CT), though less frequently employed in the evaluation of neurodegenerative diseases, remains valuable for ruling out alternative causes such as tumors, hemorrhages, or vascular lesions contributing to cognitive impairment. CT is more accessible and faster than MRI, making it suitable for acute settings or for patients with contraindications to MRI, such as those with metal implants. However, its lower resolution and inability to detect subtle neurodegenerative changes limit its diagnostic utility in chronic conditions [30].

#### 2.3.2. Functional and Molecular Imaging (PET and fMRI)

Functional and molecular imaging techniques such as Positron Emission Tomography (PET) and functional MRI (fMRI) provide critical insights into the metabolic and molecular pathology of neurodegenerative diseases. PET imaging is particularly valuable for visualizing disease-specific molecular processes. Amyloid-PET and tau-PET are highly specific for AD disease, enabling the identification of amyloid plaques and tau tangles that are hallmark features of the disease [56]. Amyloid-PET imaging, for instance, uses tracers such as florbetapir and florbetaben to detect β-amyloid deposits, offering a reliable tool for distinguishing AD from other dementias with overlapping clinical presentations, such as FTD [57,58]. Similarly, tau-PET imaging employs tracers like flortaucipir to visualize neurofibrillary tangles, providing complementary information about disease progression [59]. Dopamine transporter (DaT) PET is an essential tool for identifying dopaminergic deficits in the basal ganglia, facilitating the differentiation of PD and LBD from other parkinsonian syndromes. DaT-PET imaging has proven particularly effective in distinguishing PD from essential tremor (ET), as ET typically shows normal dopamine transporter uptake, unlike the reduced uptake observed in PD [60]. In Huntington’s disease, PET imaging can reveal early metabolic dysfunction in the striatum, even before structural atrophy becomes apparent, offering a window for preclinical diagnosis [61].

Functional MRI (fMRI) complements PET by assessing disruptions in brain networks, such as the default mode network (DMN) in AD and motor networks in PD [28,62]. This technique provides valuable insights into neuronal activity and connectivity, offering a deeper understanding of disease mechanisms.

Despite their advantages, PET and fMRI also have significant limitations. PET imaging requires the use of radioactive tracers, which are costly and not universally available, limiting widespread clinical application. The resolution of PET imaging, while sufficient for detecting molecular changes, is lower than that of MRI, potentially missing fine structural details. fMRI, on the other hand, is highly sensitive to patient-specific variability and relies on indirect measures of neuronal activity through blood oxygenation, which can lead to inconsistent findings. Additionally, both PET and fMRI have limited capacity to distinguish overlapping pathological features in complex diseases, underscoring the need for multimodal imaging approaches to achieve comprehensive and accurate diagnoses.

### 2.4. Limitations of Current Diagnostic Methods

**Figure 2:**
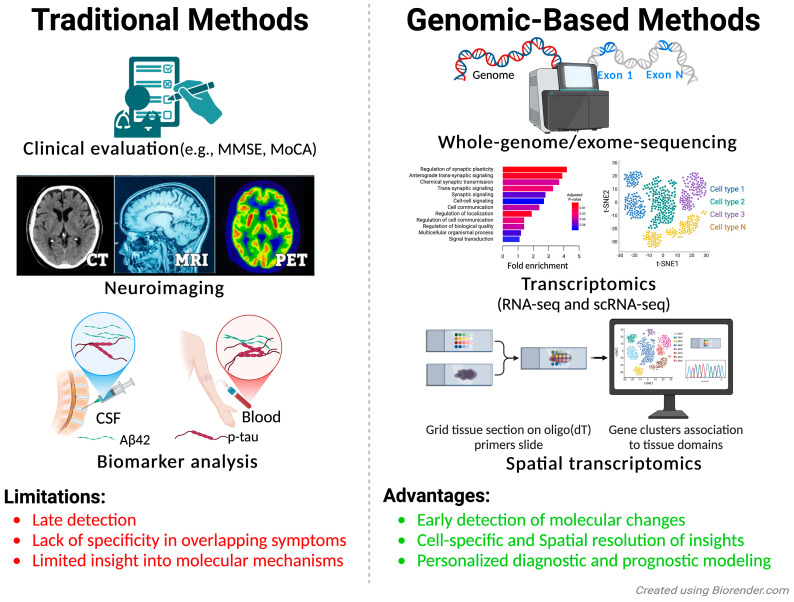
Comparison of traditional and genomic-based methods for diagnosis and prognosis of neurodegenerative diseases. This figure compares traditional diagnostic approaches and modern genomic-based methods in the context of neurodegenerative diseases. Traditional methods (left): These include clinical evaluations (e.g., MMSE and MoCA), neuroimaging (e.g., CT, MRI, and PET), and biomarker analysis of cerebrospinal fluid (CSF) and blood (e.g., Aβ42 and p-tau). While widely used, these methods are limited by late detection, lack of specificity due to overlapping symptoms, and minimal insights into the molecular mechanisms driving disease progression. Genomic-based methods (right): These advanced approaches leverage whole-genome and whole-exome sequencing (WGS and WES) to identify genetic risk variants and transcriptomics technologies (e.g., RNA-seq, scRNA-seq, and spatial transcriptomics) to uncover cell-specific and spatially resolved molecular changes. These methods enable early detection of disease-related molecular changes, provide deeper insight into cell-specific vulnerabilities, and support personalized diagnostic and prognostic modeling.

The diagnosis and prognosis of neurodegenerative diseases currently rely on a combination of clinical evaluations, biomarkers, and neuroimaging techniques. Despite advancements in diagnostic approaches, several limitations hinder the early detection and precise diagnosis of neurodegenerative diseases. Most methods, such as structural imaging or clinical assessments, identify disease only after significant neuronal damage has occurred, narrowing the window for effective intervention. Moreover, the overlapping structural and functional changes observed in different neurodegenerative diseases complicate differential diagnosis. Biomarker analysis, while valuable, often requires invasive procedures like CSF collection, which may not be feasible or acceptable for all patients. Furthermore, current diagnostic tools lack the sensitivity and specificity needed to detect early, preclinical stages of disease or differentiate between coexisting pathologies, underscoring the need for innovative and less invasive diagnostic solutions (Figure 2 and Table 2). The integration of advanced technologies, such as genomics and next-generation biomarkers, holds promise for addressing these challenges and improving early diagnosis, personalized treatment, and outcome prediction.

## 3. Harnessing Genomic Technologies for Improved Diagnosis and Prognosis of Neurodegenerative

Advancements in genomic technologies have revolutionized the diagnosis and prognosis of neurodegenerative diseases by uncovering molecular mechanisms, identifying disease-associated biomarkers, and enabling the development of personalized therapeutic strategies. From bulk genomic sequencing to single-cell and spatial transcriptomics, these tools provide complementary insights into disease pathophysiology.

### 3.1. Next-Generation Sequencing: WGS and WES

Next-generation sequencing (NGS) has emerged as a transformative technology in the field of genomics, enabling high-throughput sequencing of both DNA and RNA. Among the NGS approaches, whole-genome sequencing (WGS) provides a comprehensive analysis of the entire genome, including coding and non-coding regions, offering valuable insights into the genetic landscape of neurodegenerative diseases. For instance, WGS has been instrumental in identifying rare variants and structural abnormalities in complex diseases such as AD, ALS, and LBD [63,64,65].

In contrast, whole-exome sequencing (WES) focuses on the protein-coding regions of the genome, where the majority of known disease-causing mutations reside. This targeted approach has proven effective in identifying pathogenic mutations in familial neurodegenerative disorders, such as the *C9orf72* repeat expansion in ALS and frontotemporal dementia (FTD) [66]. WES continues to play a crucial role in understanding the genetic underpinnings of these diseases, offering a robust diagnostic tool for clinicians and researchers.

### 3.2. Transcriptomics Technologies

Transcriptomics provides a functional layer of genomic analysis by examining RNA expression profiles, offering insights into gene activity and dysregulation in neurodegenerative diseases. Advanced transcriptomics methods, such as RNA sequencing (RNA-seq), single-cell RNA sequencing (scRNA-seq), and Visium spatial transcriptomics, have significantly improved our ability to study neurodegenerative diseases at unprecedented resolution.

#### 3.2.1. RNA Sequencing (RNA-Seq)

RNA-seq, a high-throughput transcriptomics technology, enables the quantification of RNA expression levels, detection of alternative splicing events, and identification of RNA sequence variants. By providing a global view of transcriptome changes, RNA-seq has uncovered dysregulated pathways in multiple neurodegenerative conditions. For example, in AD, RNA-seq has highlighted altered expression of genes involved in amyloid-β metabolism (*APP* and *BACE1*), tau phosphorylation (*MAPT*), and neuroinflammation (*TREM2* and *CD33*) [67]. In Parkinson’s disease (PD), RNA-seq has uncovered changes in mitochondrial-related genes (*PINK1* and *SNCA*), autophagy pathways, and stress response networks in dopaminergic neurons [68]. Amyotrophic lateral sclerosis (ALS) studies using RNA-seq have identified dysregulated RNA-binding proteins such as *TDP-43* and *FUS*, which are crucial for RNA stability and splicing, offering insights into disease pathophysiology [69]. RNA-seq is an essential tool for identifying transcriptomic signatures of neurodegenerative diseases, offering biomarkers for diagnosis and prognosis. However, its bulk nature averages gene expression across heterogeneous cell populations, masking cell-type-specific changes. This limitation has been addressed with single-cell RNA sequencing and Visium spatial transcriptomics.

#### 3.2.2. Single-Cell RNA Sequencing (scRNA-Seq)

To address diagnostic challenges associated with cellular heterogeneity in neurodegenerative diseases, single-cell scRNA-seq has emerged as a transformative tool for transcriptomic profiling at the resolution of individual cells. This technology has been particularly valuable for the identification of cell-specific transcriptional changes that may serve as diagnostic or prognostic markers in the brain, a highly complex organ with diverse cell populations. In PD, scRNA-seq has revealed that specific subtypes of dopaminergic neurons in the substantia nigra exhibit unique transcriptional changes related to oxidative stress, mitochondrial dysfunction, and impaired proteostasis, shedding light on their selective vulnerability to degeneration [70]. In AD, scRNA-seq has identified disease-associated microglia (DAM), a subpopulation of immune cells characterized by upregulated expression of Trem2, ApoE, and inflammatory cytokines, highlighting their dual role in promoting neuroinflammation and clearing amyloid plaques [71,72]. Similarly, in HD, scRNA-seq has enabled detailed analysis of striatal medium spiny neurons, revealing transcriptional dysregulation driven by mutant huntingtin protein (*HTT*) and altered activity of supporting glial cells, such as astrocytes and microglia [73]. Furthermore, scRNA-seq has been instrumental in identifying cell-type-specific biomarkers and pathways, providing opportunities for targeted therapeutic interventions. However, a limitation of scRNA-seq is its inability to retain spatial context, which is critical for understanding the organization and interactions of cells within tissue architecture.

#### 3.2.3. Visium Spatial Transcriptomics

Visium spatial transcriptomics complements scRNA-seq by integrating transcriptomics with spatial information, enabling the mapping of gene expression to specific anatomical regions within a tissue. This approach is particularly valuable for studying the spatial organization of cells and their molecular changes in neurodegenerative diseases, where specific brain regions are affected [74,75].

For instance, in AD, spatial transcriptomics has been used to map region-specific expression of immune-related genes in microglia surrounding amyloid plaques, shedding light on localized inflammatory responses [76,77]. Additionally, spatial transcriptomics can be overlaid with histological or imaging data to correlate gene expression with pathological features such as amyloid plaques, Lewy bodies, or tau tangles. In AD, this integration has been particularly insightful, as spatial transcriptomics combined with amyloid and tau imaging has identified regions of heightened transcriptional dysregulation, advancing our understanding of how localized molecular changes contribute to disease progression [78,79]. Furthermore, spatial transcriptomics has revealed transcriptional differences across cortical layers involved in memory and cognition, highlighting how distinct regions of the brain contribute to disease pathology. In PD, Visium has identified spatially distinct populations of dopaminergic neurons and glial cells within the substantia nigra, providing insights into localized neuroinflammation and degeneration [80]. Similarly, in ALS, spatial transcriptomics has revealed spatially restricted patterns of neuroinflammation and excitotoxicity in motor neurons and astrocytes in the spinal cord, offering new perspectives on how localized changes drive disease progression [81].

By integrating spatial context, Visium adds a new dimension to transcriptomic studies, enabling researchers to correlate molecular changes with structural and functional alterations in the brain (Figure 2).

#### 3.2.4. Integration and Comparative Analysis

When comparing these transcriptomic technologies, each offers unique strengths and limitations. The combined use of RNA-seq, scRNA-seq, and Visium addresses the multifaceted nature of neurodegenerative diseases. RNA-seq provides a global view of dysregulated pathways, offering initial diagnostic clues. scRNA-seq refines these findings by pinpointing cell-specific changes, identifying unique cellular subtypes, and uncovering novel biomarkers. Spatial transcriptomics, such as Visium, adds a spatial dimension, correlating these molecular insights with disease-specific brain regions. The integration of these technologies offers a comprehensive framework for studying neurodegenerative diseases [82]. For example, Cummings et al. (2019) demonstrated that combining RNA-seq with genomic sequencing is highly effective for diagnosing Mendelian disorders by identifying aberrant splicing, allele-specific expression, and outlier transcript levels. This approach can highlight the downstream effects of repeat expansions or pathogenic variants in non-coding regions, facilitating their identification and functional interpretation [83]. Furthermore, in AD, the combined application of transcriptomic technologies has enabled the identification of amyloid- and tau-related transcriptional changes localized to specific cortical layers, enhancing our understanding of disease mechanisms [84]. Similarly, in ALS, the combined analysis has unraveled how motor neurons and surrounding glial cells interact spatially and molecularly to drive disease progression [85]. Such integration not only enhances diagnostic precision but also provides prognostic insights by correlating molecular and spatial dynamics with clinical outcomes. By offering molecular, cellular, and spatial insights, they bridge the gap between research and clinical application, enabling earlier and more accurate diagnosis, better prediction of disease outcomes, and more personalized therapeutic strategies.

### 3.3. Epigenomic Technologies

The advent of epigenomic technologies has opened new avenues for enhancing the diagnosis and prognosis of neurodegenerative diseases. DNA methylation patterns, such as hypermethylation of the APP promoter in AD, are emerging as non-invasive biomarkers detectable in peripheral blood or cerebrospinal fluid, offering the potential for early and accurate diagnosis [86,87]. Similarly, histone modifications, including acetylation and methylation, regulate chromatin accessibility and transcriptional activity in genes critical for neuronal function. Changes in these modifications have been shown to predict the rate of cognitive decline in AD or motor symptom progression in PD, demonstrating their value in prognostic modeling [88]. Advanced tools like single-cell ATAC-seq allow for the identification of cell-specific epigenomic changes in heterogeneous brain tissues, while emerging spatial epigenomics technologies promise to map these alterations to specific brain regions. Integration of epigenomic data with genomic sequencing, such as combining whole-genome sequencing with methylation profiling, further improves diagnostic precision by identifying epigenetically regulated pathways missed by genetic studies alone [26,89]. Furthermore, epigenomic biomarkers are being leveraged to stratify patients for targeted therapies; for example, histone modification profiles can predict responsiveness to HDAC inhibitors, paving the way for personalized medicine approaches [90]. The reduced cost and scalability of epigenomic sequencing, coupled with machine learning tools for data interpretation, are poised to make these technologies accessible in clinical settings, marking a significant step toward transforming the diagnostic and prognostic landscape for neurodegenerative diseases.

### 3.4. Multi-Omics Approaches

The integration of multi-omics approaches, encompassing genomics, transcriptomics, proteomics, and metabolomics, has emerged as a powerful strategy for uncovering the complexities of neurodegenerative diseases. By bridging distinct biological layers, these approaches offer a comprehensive view of how genetic and epigenetic variations cascade into functional and phenotypic changes, thereby advancing diagnostic and prognostic capabilities.

Proteomics, which focuses on profiling protein expression, post-translational modifications, and protein-protein interactions, has proven invaluable in neurodegenerative disease research. For instance, mass spectrometry-based proteomic studies have identified disease-specific aggregated proteins such as β-amyloid and tau in AD, as well as α-synuclein in PD [91,92]. These findings not only enhance our understanding of disease pathology but also provide biomarkers that can aid in early diagnosis and monitoring disease progression. In addition, proteomics has revealed dysregulated pathways such as impaired synaptic function and mitochondrial bioenergetics, offering potential therapeutic targets [93].

Metabolomics, which analyzes small-molecule metabolites, complements proteomics by providing insights into altered metabolic pathways underlying neurodegeneration. For example, in PD, metabolomic studies have identified perturbations in dopamine metabolism, oxidative stress markers, and mitochondrial dysfunction [94]. In AD, alterations in lipid metabolism, including reduced levels of plasmalogens and increased ceramides, have been correlated with cognitive decline [95,96]. Such metabolic changes not only serve as biomarkers but also shed light on systemic processes contributing to disease pathogenesis.

**Figure 3:**
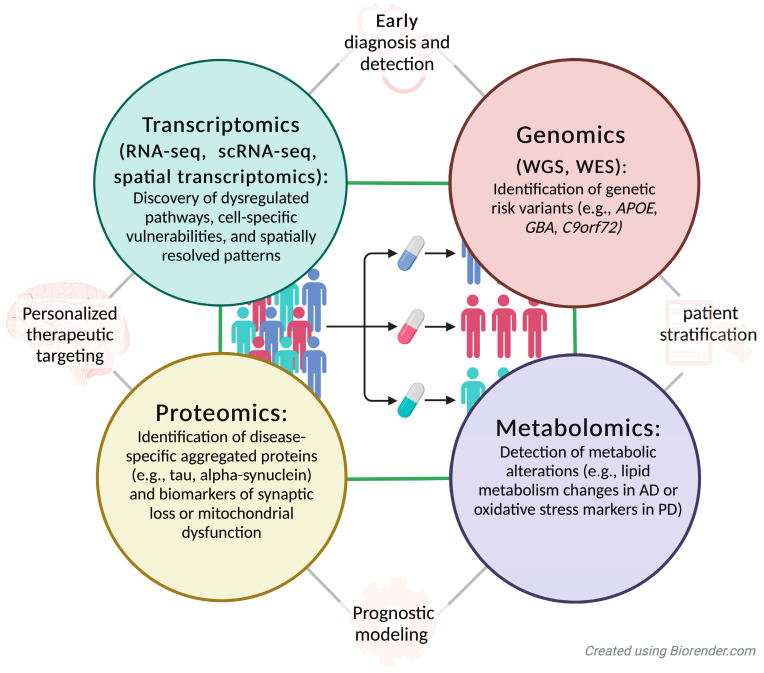
Integration of multi-omics approaches for diagnosis, prognosis, and personalized therapeutics in neurodegenerative diseases. This figure illustrates the integration of multi-omics approaches—genomics, transcriptomics, proteomics, and metabolomics—to advance the understanding, diagnosis, prognosis, and treatment of neurodegenerative diseases. Genomics (e.g., WGS and WES) identifies genetic risk variants, such as APOE, GBA, and C9orf72, that inform disease susceptibility and progression. Transcriptomics techniques (e.g., RNA-seq, scRNA-seq, and spatial transcriptomics) reveal dysregulated pathways, cell-specific vulnerabilities, and spatially resolved expression patterns. Proteomics focuses on identifying disease-specific aggregated proteins (e.g., tau, α-synuclein) and biomarkers of synaptic or mitochondrial dysfunction. Metabolomics detects metabolic alterations, such as lipid metabolism changes in Alzheimer’s disease (AD) or oxidative stress markers in Parkinson’s disease (PD). Together, these approaches enable early diagnosis, patient stratification, prognostic modeling, and personalized therapeutic targeting, paving the way for precision medicine in neurodegenerative diseases.

The integration of transcriptomics with proteomics and metabolomics has provided critical insights into how genetic and epigenetic changes translate into cellular dysfunction. For example, studies combining transcriptomics and proteomics in PD have revealed discrepancies between mRNA and protein expression levels, highlighting the role of post-transcriptional regulatory mechanisms [97]. Similarly, integrating metabolomics with transcriptomics has uncovered links between specific gene expression profiles and altered metabolic pathways in HD and ALS [98,99]. These multi-omics approaches allow researchers to identify interdependencies between different biological layers, offering a systems-level understanding of disease progression (Figure 3).

Beyond identifying biomarkers and disease mechanisms, multi-omics approaches hold great promise for advancing personalized medicine in neurodegenerative diseases. By characterizing individual patient profiles across multiple omics layers, researchers can stratify patients into subgroups based on molecular signatures, enabling more accurate prognostic predictions and tailored therapeutic interventions. For example, combining proteomic and metabolomic data can help identify patients likely to respond to specific treatments, such as mitochondrial-targeted therapies or anti-aggregation compounds. Furthermore, the integration of multi-omics data with machine learning algorithms has demonstrated remarkable potential in improving diagnostic accuracy and predicting disease progression. For instance, AI-driven analyses of combined transcriptomic and proteomic data have been used to predict conversion from mild cognitive impairment to AD with high sensitivity and specificity.

As these multi-omics approaches continue to evolve, their scalability, reduced costs, and integration into clinical workflows are expected to transform the landscape of neurodegenerative disease diagnosis and prognosis. By enabling a more holistic understanding of the molecular underpinnings of these diseases, multi-omics approaches not only deepen our understanding of disease mechanisms but also pave the way for novel diagnostic tools and precision medicine strategies.

## 4. Functional Genomics and Genetic Technologies Driving Future Prognosis

### 4.1. Insights from Functional Genomics into Disease Mechanisms and Prognostic Applications

Functional genomics has revolutionized our understanding of neurodegenerative diseases, not only by elucidating the mechanisms underlying these conditions but also by revealing molecular pathways that influence disease progression and severity. The advent of CRISPR-based genomic technologies has allowed for high-throughput screens to identify genetic modifiers of disease progression. For example, in ALS, CRISPR screens have identified *ATXN2* as a key regulator of *TDP-43* toxicity, linking its expression to the rate of motor neuron loss. Similarly, in HD, manipulation of *HTT* CAG repeat expansions through CRISPR has enabled researchers to pinpoint pathways that determine both the age of onset and progression rate, offering actionable targets for therapeutic intervention and prognostic modeling.

Gene expression studies are also integral to understanding disease-specific progression. Transcriptomic analyses in AD have highlighted distinct disease stage-specific transcriptional changes. For instance, late-stage AD is characterized by increased expression of *TREM2* and *CD33*, two genes involved in microglial activation and inflammation, correlating with rapid cognitive decline and neuronal loss [100]. Similarly, in PD, functional genomic studies of *PINK1* and *PRKN* have demonstrated that variants impairing mitochondrial quality control predict faster motor symptom progression [101]. These findings underscore the potential for linking gene expression data to clinical outcomes, enabling the identification of molecular markers that can serve as prognostic tools.

### 4.2. Genome-Wide Association Studies (GWAS) for Risk Stratification and Prognostic Insights

Beyond identifying genetic risk factors, GWAS have become instrumental in defining the prognostic landscape of neurodegenerative diseases. In AD, for example, *APOE ε4*, a major risk allele, not only elevates disease risk but also correlates with faster amyloid deposition and earlier onset of cognitive symptoms [29]. Furthermore, GWAS loci such as *BIN1* and *CLU* have been associated with the progression of tau pathology, enhancing the precision of patient stratification based on expected disease trajectories [102].

In HD, GWAS studies have uncovered modifiers such as *MLH1* and *FAN1*, which influence the age of disease onset and severity. These modifiers have been incorporated into predictive models to provide more accurate prognostic predictions for patients with *HTT* expansions [103,104]. Similarly, in PD, genetic variants in *GBA* have been linked to more aggressive disease phenotypes, including an elevated risk of cognitive impairment and dementia [105]. By contrast, *LRRK2* mutations, while associated with milder motor symptoms, exhibit variability in non-motor outcomes [106]. These examples demonstrate how GWAS findings can inform patient-specific prognostic models and guide clinical decision-making.

### 4.3. Single-Cell Genomics and Spatial Transcriptomics for Prognostic Biomarker Discovery

Advancements in scRNA-seq and spatial transcriptomics are providing unprecedented granularity in understanding neurodegenerative disease progression. scRNA-seq has revealed disease-specific cellular vulnerabilities, such as the identification of disease-associated microglia (DAM) in AD [71,107]. These microglial subtypes are linked to amyloid progression and synaptic loss, correlating with clinical markers of cognitive decline [108]. In PD, scRNA-seq has uncovered neuronal subpopulations exhibiting early transcriptional changes in mitochondrial and lysosomal pathways, offering potential prognostic biomarkers for disease severity [109].

Spatial transcriptomics further enriches our ability to map disease progression at the tissue level. In HD, spatial analyses have identified progressive transcriptional changes across affected brain regions, correlating these with motor and cognitive impairments. By integrating single-cell and spatial data, researchers are developing robust prognostic tools that track disease progression at both the cellular and regional levels, offering new avenues for monitoring and intervention [110].

### 4.4. Emerging Genetic Technologies for Prognostic Applications

Emerging genetic technologies are poised to redefine prognostic approaches in neurodegenerative diseases. Polygenic risk scores (PRS), which aggregate the effects of multiple genetic variants, are being tailored to predict not only disease risk but also progression rates. For instance, PRS models for AD that incorporate *APOE ε4* alongside other risk loci have demonstrated the ability to stratify patients based on their likelihood of rapid cognitive decline [111]. In ALS, PRS studies have been employed to differentiate patients likely to experience fast versus slow disease progression, aiding in personalized care planning and clinical trial design [112].

Epigenomic profiling is also advancing the field of prognostics. In PD, hypermethylation of the SNCA promoter, encoding α-synuclein, has been linked to accelerated disease progression, while histone acetylation patterns in HD have been associated with disease severity. The integration of epigenomic data with genomic and clinical information holds great promise for refining predictive models [113].

### 4.5. Integrating Genomics with Therapeutic Strategies to Improve Prognostic Precision

Genomic discoveries are not only illuminating disease mechanisms but also driving the development of targeted therapies that influence prognostic outcomes. Recent advancements in omics technologies have enabled the development of therapeutic approaches such as antisense oligonucleotides (ASOs), viral vector-mediated gene delivery, and gene-editing tools like CRISPR-Cas9. These therapies are leveraging genomic and transcriptomic insights to target specific pathways implicated in neurodegenerative diseases.

For instance, in ALS, ASO therapies targeting *SOD1* and *C9orf72* mutations have shown promise in extending survival and delaying symptom onset [114]. Similarly, approaches are being explored for HD, where ASOs targeting mutant HTT transcripts aim to mitigate toxic protein accumulation [115]. Additionally, viral vector-based gene therapies, such as adeno-associated virus (AAV)-mediated delivery of therapeutic genes, are showing potential in restoring lysosomal function in patients with PD with *GBA* mutations [116].

CRISPR-Cas9 technology represents another frontier, with the potential to correct disease-causing mutations in inherited forms of AD, ALS, and PD [117]. These emerging therapeutic strategies not only offer avenues to slow or halt disease progression but also provide critical data on the biological pathways driving neurodegeneration, thereby refining prognostic models.

These therapeutic advances underscore the symbiotic relationship between genomic research and clinical applications. By linking treatment strategies to genomic findings, researchers are developing prognostic models that integrate genetic, therapeutic, and clinical data, enabling personalized approaches to patient management and improving long-term outcomes.

## 5. Conclusions and Perspectives

### 5.1. Genomics Transforming Diagnosis and Prognosis

Advancements in genomic technologies have brought transformative progress in the diagnosis and prognosis of neurodegenerative diseases. Traditional methods like clinical evaluations and neuroimaging have been limited in detecting disease only after significant neuronal damage. Similarly, prognostic tools lacked the ability to predict individual disease trajectories. The advent of next-generation sequencing (NGS), including whole-genome sequencing (WGS) and whole-exome sequencing (WES), has revolutionized the field, allowing the identification of genetic variants and mutations linked to disease risk, onset, and progression. For instance, genetic markers such as C9orf72 in ALS, APOE ε4 in AD, and HTT in HD have provided unprecedented accuracy in diagnosing both familial and sporadic cases. Additionally, studies integrating genetic modifiers like FAN1 and MLH1 into predictive models for HD have significantly improved the stratification of patients based on disease trajectories [118,119]. These advancements have uncovered new pathways influencing disease severity and have set the stage for personalized interventions.

Transcriptomics technologies, including RNA-seq, scRNA-seq, and spatial transcriptomics, further complement genomic approaches by offering insights into cell-type-specific vulnerabilities and disease-stage-specific molecular signatures. For example, the identification of disease-associated microglia (DAM) in AD and spatially resolved transcriptomic patterns in ALS has added critical layers of understanding to the molecular underpinnings of disease progression [120,121]. These breakthroughs enable researchers and clinicians to move beyond traditional diagnostic limitations, achieving systems-level perspectives of neurodegenerative disorders.

### 5.2. Overcoming Traditional Limitations

Genomic approaches have addressed several limitations inherent in traditional diagnostic and prognostic methods. First, they enable early detection and diagnosis. Genetic risk variants such as APOE ε4 in AD and GBA in PD, combined with transcriptomic signatures of early neuroinflammation or synaptic dysfunction, allow detection of molecular changes well before clinical symptoms appear. Technologies like scRNA-seq also uncover subtle, pre-symptomatic alterations in neuronal and glial populations, enabling earlier intervention.

Second, these methods improve prognostic precision by identifying markers that stratify patients based on disease trajectory. For instance, MLH1 and FAN1 in HD predict disease severity [122], while GBA mutations in PD correlate with rapid cognitive decline [123]. Polygenic risk scores (PRS) and multi-omics integration further enhance prediction accuracy, enabling personalized care plans. Finally, by uncovering dysregulated pathways like tau phosphorylation in AD or lysosomal dysfunction in PD, genetic technologies provide insights into molecular mechanisms, offering actionable therapeutic targets that traditional tools fail to address.

### 5.3. Future Opportunities in Prognosis and Patient Care

Genomics and transcriptomics are opening exciting new avenues for patient care. One major opportunity lies in the development of AI-driven prognostic models. By integrating multi-omics, clinical, and imaging data, artificial intelligence and machine learning will enable precise prediction of individual disease trajectories, guiding personalized treatment strategies. For instance, molecular abnormalities such as *TREM2* expression in microglial subpopulations and elevated CSF p-tau levels in AD have been correlated with disease progression and specific clinical outcomes. *TREM2* expression is associated with microglial activation and β-amyloid plaque clearance, while elevated CSF p-tau levels strongly correlate with tau pathology and disease staging [124,125]. Incorporating such molecular findings with clinical and imaging data through AI-powered analysis could stratify patients into subpopulations, refine diagnosis and staging, and inform targeted therapeutic interventions. Similarly, in PD, *GBA* mutations that impair lysosomal function have been associated with an increased risk of disease onset and progression [126]. These insights provide a foundation for personalized medicine, where therapies such as lysosomal enhancers for patients with PD with GBA mutations or *TREM2*-targeted therapies in patients with AD with microglial dysfunction represent tailored interventions based on individual genetic profiles.

Furthermore, earlier detection of genetic risks through multi-omics approaches will support preventive strategies, such as lifestyle modifications or pre-symptomatic therapies, to delay disease onset and progression. Advances in single-cell and spatial transcriptomics will also refine monitoring and therapeutic targeting by revealing cell-specific and region-specific biomarkers of disease. For instance, the application of scRNA-seq and Visium in brain samples has identified distinct molecular signatures of dysfunctional microglia and neurons, which may act as actionable targets for therapy [79,82,127].

The integration of such molecular findings into predictive AI models has the potential to revolutionize clinical care for neurodegenerative disorders. By leveraging these technologies, clinicians can better anticipate disease trajectories, assess the effectiveness of interventions, and ultimately improve patient outcomes. While challenges such as data interpretation complexity, heterogeneity of neurodegenerative disorders, and accessibility of advanced technologies remain, the ongoing development of AI-based frameworks provides an unprecedented opportunity to overcome these barriers and advance personalized care.

### 5.4. Collaborative Efforts and Continued Research

To fully realize the potential of genomic technologies in clinical practice, collaborative efforts among researchers, clinicians, and policymakers are essential. Standardized protocols for genomic data collection, analysis, and reporting must be developed to ensure consistency and reliability. Moreover, expanding genomic studies to include diverse populations is crucial for ensuring equitable access and applicability of findings.

Investment in infrastructure and workforce training is equally important to integrating genetic tools into clinical workflows. By fostering multidisciplinary collaboration, the field can accelerate the translation of these advancements into routine patient care, ultimately improving outcomes for individuals with neurodegenerative diseases.

### 5.5. Closing Remarks

The integration of advanced genetic approaches, from sequencing technologies to dynamic transcriptomics, represents a paradigm shift in the diagnosis and prognosis of neurodegenerative diseases. By enabling earlier detection, precise prognostic predictions, and deeper mechanistic insights, these innovations are addressing long-standing limitations of traditional methods. As the field continues to evolve, the combination of genomics, transcriptomics, and AI-driven analytics holds the promise of transforming patient care, improving outcomes, and enhancing quality of life for individuals affected by these devastating disorders.

## Figures and Tables

**Table 1:** Genes implicated in neurodegenerative diseases and key clinical synopsis. This table summarizes key genes implicated in neurodegenerative diseases, their associated clinical features, inheritance patterns, and corresponding OMIM phenotype numbers. Genes associated with the disease are categorized by early-onset (EO), late-onset (LO), or both (EO/LO).

Disease	Gene(s)	Clinical Synopsis	Inheritance Pattern	OMIM Phenotype Number
Alzheimer’s Disease (AD)	APP (EO), PSEN1 (EO), PSEN2 (EO) [22,23], APOE ε4 (LO) [26]	Progressive memory loss, cognitive decline, language and visuospatial deficits, β-amyloid plaque accumulation, neurofibrillary tangles	Autosomal dominant	104300
Parkinson’s Disease (PD)	PARK2 (EO), SNCA (EO/LO), LRRK2 (LO) [24]	Resting tremors, bradykinesia, rigidity, postural instability, Lewy bodies formation, loss and degeneration of brain tissue	Autosomal dominant/recessive	168600
Huntington’s Disease (HD)	HTT (EO) [27,28]	Chorea, cognitive impairment, psychiatric symptoms	Autosomal dominant	143100
Amyotrophic Lateral Sclerosis (ALS)	C9orf72 (EO/LO), SOD1 (EO/LO) [29]	Progressive muscle weakness, spasticity, fasciculations, respiratory failure, upper and lower motor neuron degeneration	Autosomal dominant/recessive	105400
Lewy Body Dementia (LBD)	SNCA (EO/LO), GBA (LO) [30]	Cognitive decline, visual hallucinations, Parkinsonism, Lewy body formation	Autosomal dominant	127750

**Table 2:** Diagnostic methods and their applications in neurodegenerative diseases. This table outlines various diagnostic approaches used in neurodegenerative diseases, including Alzheimer’s disease (AD), Parkinson’s disease (PD), Huntington’s disease (HD), amyotrophic lateral sclerosis (ALS), frontotemporal dementia (FTD), and Lewy body dementia (LBD). In addition to traditional methods, genetic and transcriptomic approaches are highlighted as emerging tools for early diagnosis and therapeutic target identification.

Diagnostic Method	Approach	Utility	Limitations
Clinical Evaluation	Comprehensive history taking, neurological, and physical exams.	Identifies hallmark symptoms of diseases (e.g., bradykinesia for PD, chorea for HD, and memory loss for AD).	Lacks precision for early-stage or overlapping symptoms in neurodegenerative diseases.
	Genetic testing (e.g., CAG repeat expansion in HTT for HD; SOD1 and C9orf72 mutations for ALS).	Confirms familial disease cases.	Limited availability, cost, and ethical considerations in predictive testing.
Neuropsychological Testing	Cognitive function tests (e.g., MMSE, MoCA).	Detects cognitive impairment patterns specific to diseases (e.g., memory deficits in AD, executive dysfunction in FTD).	Insufficient for detecting structural or molecular changes.
	Neurophysiological studies (e.g., EMG, nerve conduction studies).	Assesses neuromuscular diseases like ALS.	Limited diagnostic specificity for other neurodegenerative disorders.
Biomarker Analysis	CSF biomarkers (e.g., Aβ42, t-tau, p-tau for AD; α-synuclein for PD and LBD).	Detects molecular changes.	Invasive collection methods; overlapping markers across diseases.
	Blood biomarkers (e.g., plasma Aβ, tau, NfL).	Less invasive, scalable, and potential for early diagnosis.	Requires further validation for sensitivity and specificity.
Neuroimaging	Structural imaging (e.g., MRI: hippocampal atrophy in AD; CT: rule out alternative causes like tumors or hemorrhages).	Identifies structural and molecular changes in the brain.	Limited sensitivity for early molecular changes; MRI is costly and time-consuming.
	Functional imaging (e.g., PET: tau imaging for AD, DT-PET for PD).	Provides insights into network disruptions (e.g., fMRI for AD and PD).	Expensive, limited availability (radioactive tracers for PET), and variability in results.
Genetic and Transcriptomic	Next-generation sequencing (NGS), microarray analysis	Identifies genetic variants (e.g., APOE ε4 for AD, TARDBP for ALS).	High cost and technical expertise required; limited to research or specialized centers.
	Transcriptomic profiling (e.g., RNA sequencing).	Helps understand disease-specific pathways, aiding in early diagnosis and targeted treatment.	Requires integration with other diagnostic tools for clinical applicability.

## Supplementary Materials

The following supporting information can be downloaded at: https://www.mdpi.com/article/10.3390/genes16020135/s1, Figure S1.
