# Supplementary figures and images for: Genomic and Transcriptomic Approaches Advance the Diagnosis and Prognosis of Neurodegenerative Diseases
Source: Genes (Basel). 2025 Jan 24;16(2):135. doi: 10.3390/genes16020135 (PMC11855287; doi:10.3390/genes16020135)

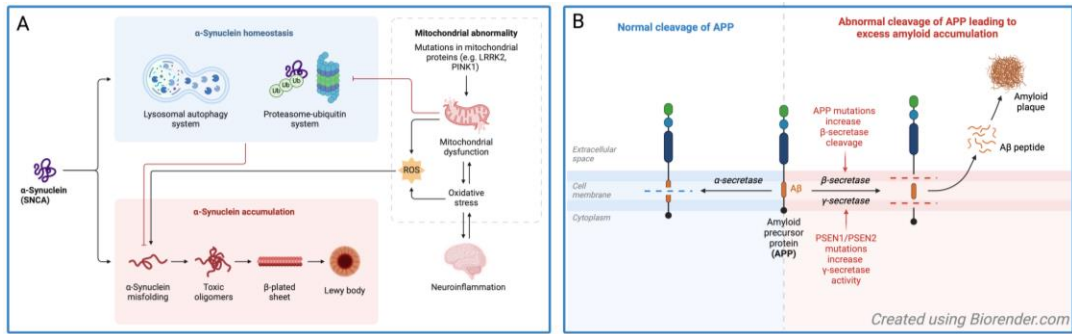

Figure S1

Supplement: Supplementary file 1 [file genes-16-00135-s001.zip › genes-3415773-supplementary.pdf]
